# Sphingolipid Metabolism and Signaling in Skeletal Muscle: From Physiology to Physiopathology

**DOI:** 10.3389/fendo.2020.00491

**Published:** 2020-08-07

**Authors:** Sophie Tan-Chen, Jeanne Guitton, Olivier Bourron, Hervé Le Stunff, Eric Hajduch

**Affiliations:** ^1^Centre de Recherche des Cordeliers, INSERM, Sorbonne Université, Université de Paris, Paris, France; ^2^Institut Hospitalo-Universitaire ICAN, Paris, France; ^3^Université Saclay, CNRS UMR 9197, Institut des Neurosciences Paris-Saclay, Orsay, France; ^4^Assistance Publique-Hôpitaux de Paris, Département de Diabétologie et Maladies Métaboliques, Hôpital Pitié-Salpêtrière, Paris, France

**Keywords:** ceramide, insulin, diabetes, obesity, sphingosine-1-phosphate, glycosphingolipids, sphingomyelin

## Abstract

Sphingolipids represent one of the major classes of eukaryotic lipids. They play an essential structural role, especially in cell membranes where they also possess signaling properties and are capable of modulating multiple cell functions, such as apoptosis, cell proliferation, differentiation, and inflammation. Many sphingolipid derivatives, such as ceramide, sphingosine-1-phosphate, and ganglioside, have been shown to play many crucial roles in muscle under physiological and pathological conditions. This review will summarize our knowledge of sphingolipids and their effects on muscle fate, highlighting the role of this class of lipids in modulating muscle cell differentiation, regeneration, aging, response to insulin, and contraction. We show that modulating sphingolipid metabolism may be a novel and interesting way for preventing and/or treating several muscle-related diseases.

## Introduction

Sphingolipids (SLs) have been studied and described for several years as modulators of many functions in cells, including proliferation, differentiation, mobility, and survival. Skeletal muscle represents by far the most important tissue for maintaining posture/vertical position, movement, and locomotion of the human body. It is also the major tissue where insulin stimulates glucose uptake, and to store it into glycogen, making it a key organ in carbohydrate homeostasis maintenance (1, 2). Since abundant literature shows that SLs regulate positively or negatively many of biological functions, such as cell proliferation and differentiation, contraction, and insulin response, the extensive study of the function of these lipids in muscle cells is very important because they could represent pharmacological targets to counteract several important diseases, such as muscular dystrophies and diabetes.

## Overview of Sphingolipid Metabolism

SLs represent one of the major classes of eukaryotic lipids. They were originally described as intermediate lipids for the synthesis of other lipids. They mainly play a structural role, especially in cell membranes where they exert signaling properties and are capable of modulating multiple cell functions, such as apoptosis, cell proliferation, and inflammation (3). This is a class of lipids defined by their sphingoid skeleton made up of 18 carbons with an amine and two alcohol groups (4). This lipid class includes many lipids with various structures and functions.

**Figure 1:**
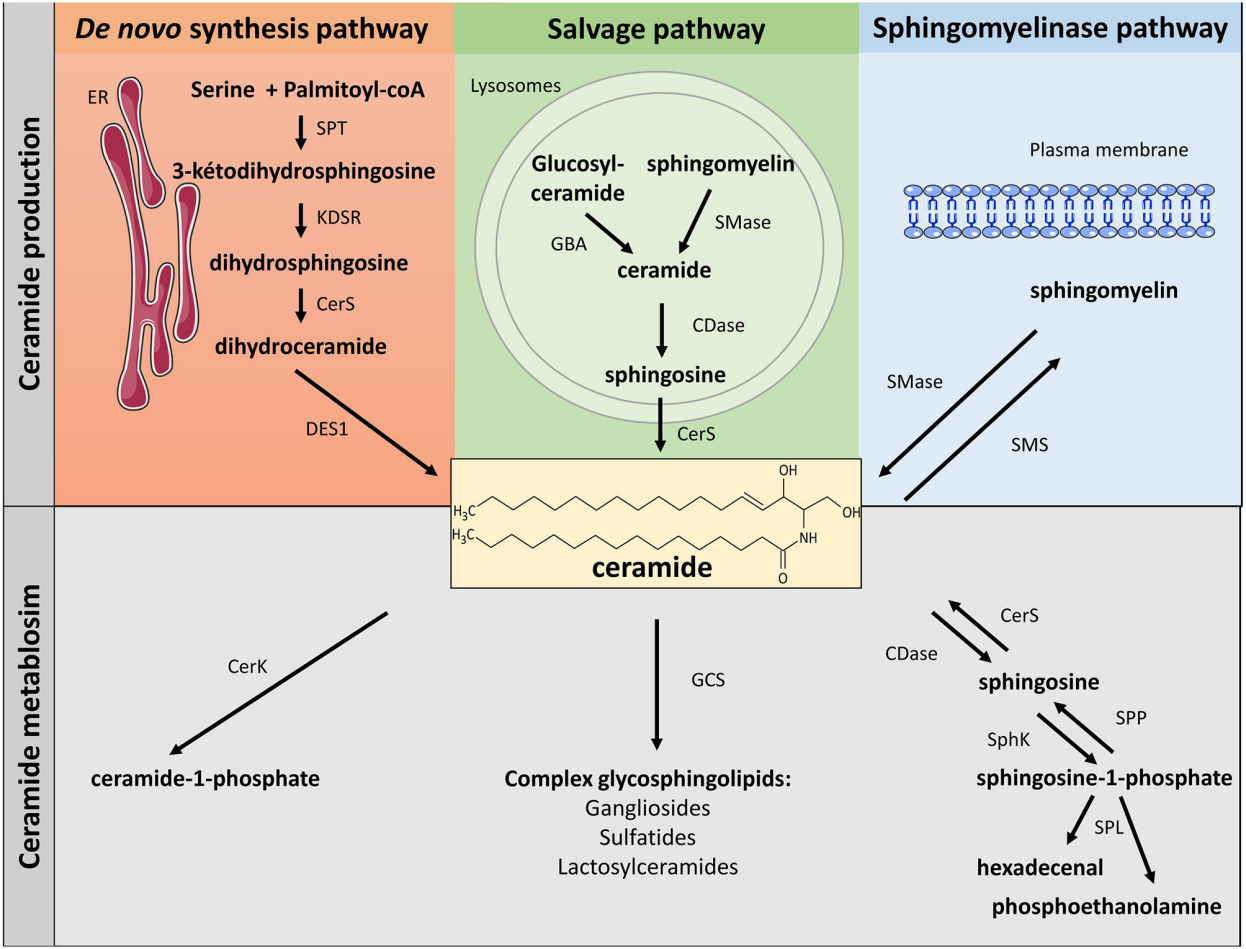
Metabolism of sphingolipids. SLs can be synthetized through three major metabolic pathways: the *de-novo* pathway coming from the condensation of SFA (palmitate) with serine, the salvage pathway and the SMase pathway. ER, endoplasmic reticulum; SPT, serine palmitoyl transferase; KDSR, 3-keto-dihydrosphingosine; CerS, ceramide synthase; DES, dihydroceramide desaturase; GBA, acid β-glucosidase; SMase, sphingomyelinase; CDase, ceramidase; SMase, sphingomyelinase; SMS, sphingomyelin synthase; CerK, ceramide kinase; GCS, glucosyl ceramide synthase; SphK, sphingosine kinase; SPP, lipid sphingosine phosphatase; SPL, sphingosine-1-phosphate lyase.

Ceramides, central lipids for SL biosynthesis, are mainly synthesized via three metabolic pathways (Figure 1):

- The *de-novo* synthesis pathway from saturated fatty acid (FA) that takes place in the endoplasmic reticulum (ER).
- The sphingomyelinase (SMase) pathway that uses SMase to convert sphingomyelin (SM) present in the cell membrane to give ceramide.
- The salvage pathway in the lysosome that produces sphingosine, and then ceramide after breakdown of complex SL.

### *De-novo* Ceramide Synthesis Pathway

SL biosynthesis is induced at the cytosolic leaflet membrane of the ER where ceramide is synthesized after several enzymatic reactions (5, 6). First, condensation of palmitate and serine forms 3-keto-dihydrosphingosine. This reaction is catalyzed by serine palmitoyl transferase (SPT) and is rate-limiting for the pathway. It should be noted that SPT can also use both glycine and alanine to make atypical 1-deoxysphingolipids (7). Functions of 1-deoxysphyngolipids remain still unclear, but these lipids appear to be significantly elevated in plasma from diabetic (8) and non-alcoholic fatty liver disease patients (9). In addition, 1-deoxysphingolipids have been shown to be toxic to β-cells and neurons (10, 11). SPT can also use myristate instead of palmitate, resulting in the production of a d16:0 sphingoid base (10). Interestingly, d16 SL promoted cell death of cardiomyocytes (12). Then, 3-keto-dihydrosphingosine (KDSR) is reduced to dihydrosphingosine, which is acylated by ceramide synthases (CerS) to produce dihydroceramide (Dcer). In mammals, six CerS isoforms (CerS 1 to 6) are expressed. They perform identical chemical reaction but produce ceramide species with different acyl-CoA chain length. Eventually, Dcer desaturase-1 (DES1) desaturates Dcer to give ceramide (Figure 1).

During the conversion of Dcer into ceramide, an alternative product can be synthesized, 4-hydroxyceramide, or phytoceramide (13), and it is the second member of the DES family, C4-hydroxylase/Δ4-desaturase (DES2) that catalyses the formation of phytoceramide from Dcer (13). DES2 is highly expressed in fungi, plants, but also in the intestine, kidney, and skin where phytoceramide is present in large quantity (14).

ER-synthesized ceramide is then immediately transported into the Golgi apparatus to generate other SL. This translocation is mediated by two types of ceramide transport. The first, and the most characterized ceramide transporter, is the non-vesicular transporter CERamide Transporter (CERT). CERT specifically transports ceramide from the ER and displays small activity toward other SL. CERT efficiently mediates the transfer of ceramide-containing C14–C20 FAs to the Golgi in order to be transformed into SM. The second type of ceramide transporter is a vesicular transporter (15). This transporter ensures the transport of long-chain ceramide (>C20) into the Golgi to generate glucosylceramides. Unlike CERT, this vesicular transporter has not been well-characterized and is known to be dependent on phosphoinositide-3-kinase (PI3K) activity (4).

Once in the Golgi apparatus, ceramide can be further metabolized to SM and complex glycosphingolipids.

SM, one of the major components of cell membranes, is synthesized through the transfer of a phosphorylcholine head group from phosphatidylcholine to ceramide. This reaction is catalyzed by SM synthases (SMS) (5). Two isoforms of SMS exist: SMS1 and 2. Both are present in the Golgi, but SMS2 is also localized at the plasma membrane, maintaining plasma membrane SM content (Figure 1) (16).

Ceramide can also be transformed into glycosylphingolipids, such as glucosylceramide (in the Golgi apparatus), or galactosylceramide (in the ER). Glucosylceramide is the precursor of gangliosides, whereas galactosylceramide will be transformed into sulfatides (Figure 1) (17). Glucosylceramides are synthesized from ceramide and UDP-glucose. This reaction is catalyzed by glucosylceramide synthase (GCS), which is localized on the cis side of the Golgi (17). On the other hand, synthesis of galactosylceramide takes place on the lumenal surface of the ER under the control of the galactosyl transferase (CGT) (4, 18).

Glucosylceramides are SL that play an essential role in mammal development and survival, as for instance, in cell recognition processes (19). Galactosylceramides are essential to myelin structure and function, and they are involved in oligodendrocyte function (4, 20).

In the Golgi, ceramides can also be phosphorylated by the ceramide kinase (CerK), thus generating ceramide-1-phosphate (C1P). C1P acts as a docking site for the cytosolic phospholipase A2 and enhances arachidonic acid release. C1P is involved in cell growth, anti-apoptosis, and inflammation in numerous cell types (Figure 1) (21, 22).

### Sphingomyelinase and Ceramidase Pathways

Hydrolysis of SM to give ceramide is catalyzed by SMases. SMases catalyze the cleavage of the phosphocholine head group of SM to generate ceramide (Figure 1). Three kinds of SMases have been identified, based upon their optimum pH: acid sphingomyelinase (aSMase), alkaline SMase, and neutral sphingomyelinase (nSMase) (4). Even though they catalyze a similar reaction, these three enzymes are different and display different subcellular localizations. Alkaline SMase, exclusively expressed in the intestine and liver, plays a role in the absorption of dietary SM. On the other hand, both aSMase and nSMase are ubiquitously expressed and play major roles in SM catabolism in most tissues. aSMase is predominantly a lysosomal enzyme that metabolizes SM present on endosomal membranes, whereas three isoforms of nSMase (nSMase 1, nSMase 2, and nSMase 3) are present in the inner leaflet of the plasma membrane (23). nSMase 2 is the most studied isoform and has emerged as a key mediator in the generation of ceramide in response to many stresses to disturb diverse signaling pathways in the fields of cancer, growth and development, and in inflammatory responses (24).

SM degradation is a major pathway involved in producing ceramide and some of its lipid metabolites, and in particular sphingosine-1-phosphate (S1P), last SL before the final stage of SL degradation (Figure 1). Ceramide is first deacylated by ceramidase (CDase) to produce sphingosine (4). There are three isoforms of CDase classified, like SMases, according to their optimal pH of function and their subcellular location. Acid CDase (ASAH1) is localized in lysosomes and is more effective in deacylating medium-chain ceramides (C12- and C14- rather than C16- and C18-ceramide) (4, 25). Neutral CDase (ASAH2) is an important regulator for the production of sphingosine and S1P at the plasma membrane (4, 25). There are three alkaline CDases (ACER). ACER1 is mainly localized in the epidermis ER and it deacylates C24:0- and C24:1-ceramides. ACER2 is localized in the Golgi and is very strongly expressed in the placenta. ACER2 deacylates C16-, C18-, C20-, C24:0, and C24:1-ceramides. ACER3 is a phytoceramidase that is localized in both the RE and the Golgi. It is expressed in many tissues, its most important expression being found in the placenta, like ACER2 (4, 25). Interestingly, sphingosine is considered to be exclusively generated from ceramide through the action of CDase. *De-novo* synthesis of sphingosine has been ruled out since DES1 specifically acts on Dcer, not on dihydrosphingosine (26).

Then, sphingosine is phosphorylated by sphingosine kinases (SphK) to generate S1P (Figure 1) (4). Two isoforms of SphK exist (SphK1 and SphK2). They catalyze the same reaction but with different subcellular localizations (27). SphK1 is mainly a cytosolic enzyme and contains residues that bind acidic phospholipids that contribute to SphK1 intracellular localization (28). SphK1 can be activated by growth factors, cytokines, and G protein-coupled receptors (GPCR) ligands. Extracellular signal-regulated kinase 1/2 (ERK1/2) phosphorylate SphK1 on its serine 225 residue, thus inducing its translocation to cell plasma membrane where sphingosine is localized (29). In contrast, SphK2 is localized in different cell compartments, such as the nucleus, endoplasmic reticulum, and mitochondria (27). The mechanism by which SphK2 is regulated remains obscure and, in opposite to SphK1-produced S1P that is rapidly excreted outside the cell, SphK2-produced S1P is rapidly degraded to be recycled into sphingosine to give ceramide (30).

S1P degradation is controlled either by specific lipid phosphate phosphohydrolases (LPP) that hydrolyze S1P to give sphingosine, or by the S1P-lyase (SPL), splitting S1P into hexadecenal and phosphoethanolamine (Figure 1) (31).

S1P is a strong signal mediator that affects several cellular functions essential for health and diseases (32). Many different S1P-mediated actions are explained by the fact that S1P modulates intracellular functions, but also, after secretion into the extracellular environment, S1P acts as a ligand of GPCR called S1P receptors that are present on most cell membranes (32). S1P possess five specific receptors (S1P1-5) on the cell surface (27). Binding of S1P to its receptors allows the transmission of various signals in cells. In addition to being able to relay its effects via its receptors, S1P has several direct intracellular targets involved in gene expression, inflammation, and mitochondrial function (27).

**Figure 2:**
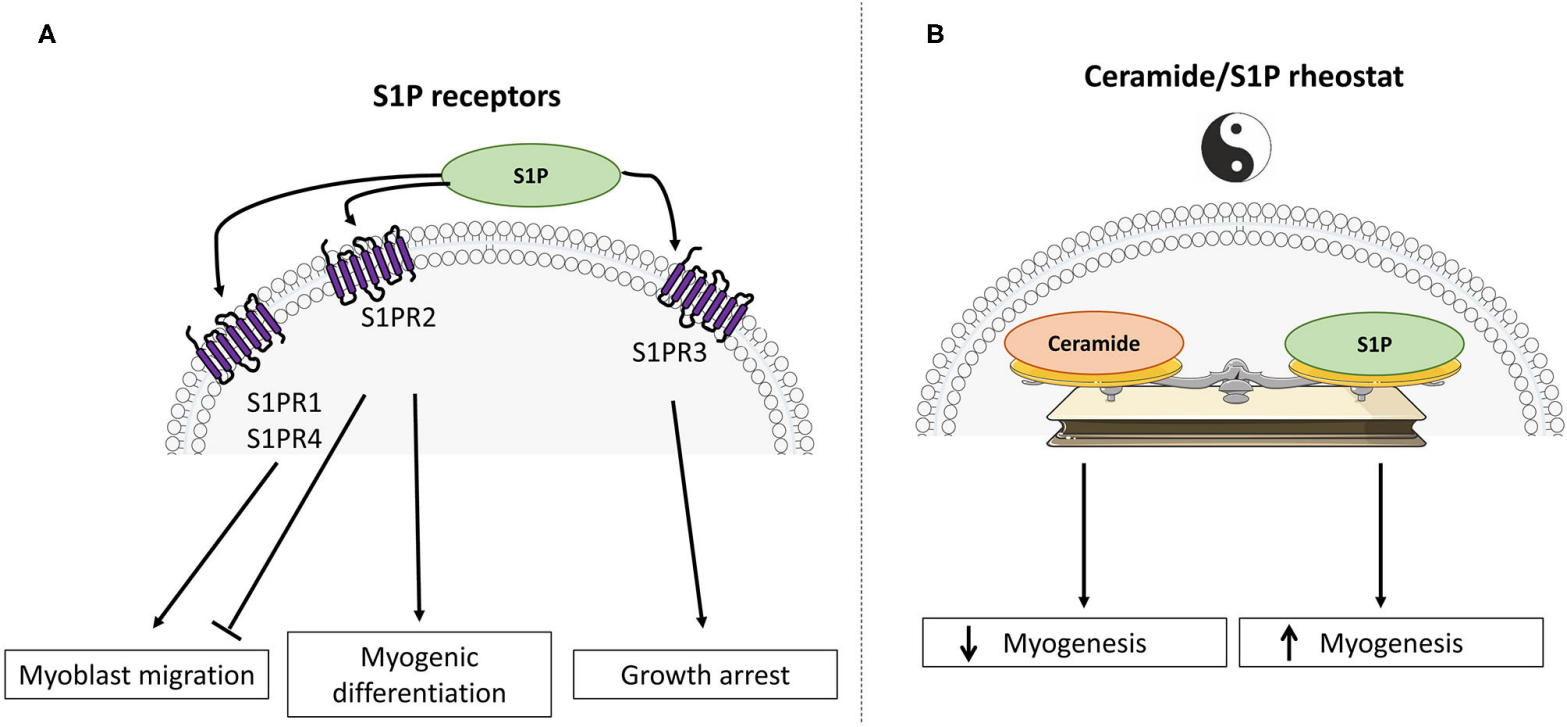
Divergent roles of sphingosine-1-phosphate and ceramide in skeletal muscle cell fate. **(A)** Schematic representation of S1PR signaling activation in muscle differentiation. **(B)** The “ceramide / sphingosine-1-phosphate rheostat” model emphasizes antagonistic roles of ceramide and S1P in the regulation of myogenesis and glucose metabolism.

Interestingly, S1P has been shown to play a significant role in the control of cell survival and growth (effects opposite to those of ceramides), and SphK1 has emerged as a central signaling enzyme. As such, SphK1 is placed at a key point, controlling the balance between the prosurvival and proapoptotic SL metabolites, namely the “sphingolipid rheostat” (Figure 2) (32).

### Salvage/Recycling Pathway

The recycling pathway for long-chain sphingoid bases leading to SL regeneration constitutes between 50 and 90% of SL biosynthesis (33, 34). This suggests a crucial role of this pathway in the biosynthesis/recycling of SL and an important implication in cell signaling.

The constitutive degradation of complex SL and glycosphingolipids (35, 36) occurs in acidic subcellular compartments such as endosomes and lysosomes. In the case of glycosphingolipids, exohydrolases (β-glucosidase acid) release monosaccharide units to generate ceramide. Likewise, SM present in the lysosomes are reconverted into ceramide via aSMase (37). The common metabolic product of these degradations (ceramide) is hydrolyzed by acid CDase to give sphingosine and free FAs capable (unlike ceramide) of leaving lysosomes (38, 39). Then, sphingosine and free FAs released from the lysosome enter the ceramide biosynthesis pathway (40, 41) to generate new ceramide molecules via CerS (42–44), or can be phosphorylated by sphingosine kinases into S1P (Figure 1) (43).

## Sphingolipids and the Regulation of Muscle Biology

In skeletal muscle, and in addition to their roles as structural membrane components, it has been shown that many metabolites derived from the metabolism of ceramide play different key roles in various biological effects, such as cell proliferation, differentiation, survival, and mobility. In addition, it is now clear that several SL play a major role in regulating muscle insulin response. Two SL derivatives have been mainly studied, namely ceramide and S1P, and it is interesting to note is that these two lipids often relay opposite biological actions. For example, ceramide is involved in apoptosis, cellular stress, and cell growth arrest, while S1P plays important roles in mitogenesis, cell differentiation, and migration. Similarly, S1P has been shown to prevent programmed cell death normally induced by ceramide. This homeostatic system is known as the ceramide/S1P rheostat (Figure 2) (32).

At the muscle level, ceramide and S1P have been also shown to exert opposite roles. Indeed, intracellular accumulation of ceramides displays largely negative actions, while an increase in S1P concentrations in muscle cells frequently potentiate opposite functions to those of ceramide.

### Regulation of Growth, Differentiation, Regeneration, and Aging of Muscle Cells by SPHINGOLIPIDS

There is a mechanism that is regulated by the ceramide/S1P rheostat in muscle cells, which is growth and differentiation of skeletal muscle cells (Figure 2).

Most of the experiments studying *in-vitro* skeletal muscle growth and differentiation have been carried out in the C2C12 muscle cell line, derived from mouse muscle satellite cells (45). They showed that S1P plays a major role in the activation of satellite cells. More specifically, inhibition of S1P synthesis through the use of SphK inhibitors demonstrated the importance of S1P to induce the entry of satellite cells into the cell cycle by inhibiting cell proliferation and inducing their myogenic differentiation (46, 47). The importance of S1P receptors in the initiation of the C2C12 muscle cell differentiation phenotype has been highlighted in this mechanism (Figure 2A) (47), and it has been demonstrated that S1P stimulates positively cell migration through the activation of both S1P1 and S1P4, whereas S1P2 was found to negatively regulate cell migration (48). In addition, activation of the S1P3 suppresses cell cycle progression in muscle satellite cells (49).

Interestingly, S1P receptor expression changes occur during myoblast differentiation into myotubes. Indeed, it has been observed a downregulation of the S1P2, while expression of the S1PR3 was increased in differentiated cells (50). Using both pharmacological and genetic approaches, a study demonstrated that S1P, through its binding to S1P2, reduced serum-induced myoblast proliferation and potently stimulates myogenesis (47).

SphK1 also plays a role in this mechanism, because overexpression of this kinase in cells reduced myoblastic proliferation and increased the expression of myogenic differentiation markers (51). In contrast, inhibition of SphK1 expression increased cell proliferation and delayed the onset of myogenesis (51, 52). In addition, stimulation of myogenesis in cells overexpressing SphK1 is inhibited when the S1P2 receptor was suppressed, reinforcing the idea that the expression of the S1P2 receptor is critical for relaying the pro-myogenic action of S1P in muscle cells (53).

In addition to muscle differentiation, S1P plays an important role in skeletal muscle regeneration. Indeed, a notable property of skeletal muscle is its capacity to regenerate in response to injury. Satellite cells are activated, proliferate, migrate, differentiate, and fuse to form new myofibers (54), and several studies have shown that S1P signaling was involved in this process [reviewed in (55)]. S1P stimulates the proliferation of a muscle cell reserve through the activation of its S1P1 (56), and exogenous addition of S1P stimulates the growth of regenerating myofibers after a myotoxic injury induced by intramuscular injection of bupivacaine (57). Interestingly, S1P was shown to suppress muscle degeneration in Duchenne muscular dystrophy (58), and to exert a positive action for activating satellite cells and muscle regeneration in dystrophic muscles (59, 60). It was also reported that S1P could protect skeletal muscle tissue against eccentric contraction-induced damage, further emphasizing the relevance of S1P signaling in skeletal muscle protection and regeneration (61).

In contrast to S1P signaling, ceramide appears to negatively regulate myogenic differentiation. It has been shown in C2C12 muscle cells that a treatment with ceramide abrogated cell differentiation (62) and the myogenic marker myogenin (63). Inhibition of the *de-novo* ceramide synthesis pathway using potent inhibitors (fumonisin B1 and myriocin) decreased intracellular concentrations of ceramide and increased the appearance of a cell differentiation phenotype (63). Interestingly, ceramide has been shown to regulate negatively muscle differentiation through downregulation of phospholipase D expression and activity (63).

Similar results were obtained after treating myoblasts with the pro-inflammatory cytokine tumor necrosis factor α (TNFα), known to induce ceramide synthesis through the activation of the SMase pathway (64). Strle et al. (65) showed that TNFα-induced ceramide production inhibited the expression of critical muscle-specific transcription factors and myogenesis. In addition, inhibition of ceramide synthesis improved myocyte size under conditions inducing muscle atrophy (a mouse model of cancer-induced cachexia and in muscle cells treated with TNF-α) (66), thus reinforcing the negative action of the lipid on muscle-mass regulation.

All these results highlight the importance of the SL metabolism in regulating muscle differentiation mechanism. Ceramide and S1P act as a rheostat by regulating myogenic differentiation of satellite cells rather than their survival, depending on cell conditions (Figure 2B).

Some data also demonstrated that SL metabolism could be involved in the development of sarcopenia with aging and muscle atrophy, often observed during obesity. Several studies showed that, during aging, there is a decrease in muscle protein synthesis (67) and increased tissue insulin resistance (68). Insulin is a powerful anabolic factor that promotes muscle protein synthesis, primarily by inhibiting protein breakdown (69, 70). A metabolic characteristic of aging is an increase of intramyocellular lipid content, and this lipid increase could play an important role in the development of insulin resistance of muscle cells with age. Studies have recently shown a significant accumulation (2- to 4-fold) of different SL, including ceramide, sphingosine, and their respective metabolites Dcer, sphingosine, and S1P in muscle cells from older mice compared to young congeners (71). In parallel, increased expression of inflammatory markers TLR2, TNF-α, and IL-1β, and a higher phosphorylation and ubiquitination of NF-κB key inhibitor IκBα were observed with aging (71). These data could demonstrate that an association exists between the accumulation of critical bioactive SL, such as ceramide, associated with inflammation, and a decrease in lean mass and muscular strength observed with age in mouse skeletal muscle. Unfortunately, no human studies corroborating these results obtained in rodents have been published so far.

Interestingly, an increase in ASMase expression has been shown to induce a decreased skeletal muscle force in mice (72). Thus, this modification ASMase expression could play in important role in changes of SL turnover observed with age (see above). Using various ASMase inhibitors, Babenko et al. (73) demonstrated that downregulation of ASMase reduced skeletal muscle ceramide content and ceramide/SM ratio and increased SM of old rats to the levels close to those observed in young animals. Unfortunately, S1P content had not been assessed in these conditions. Since S1P displays pro-differentiation activity (see above), S1P concentrations could vary inversely to those of ceramides. In addition, inhibition of ASMase activity improved skeletal muscle insulin response of old rats up to the level of younger animals (74). If confirmed in humans, these data suggest that age-dependent upregulation of ASMase could play an important role in the modulation of skeletal muscle ceramide content during age and could be a therapeutic target for treatment of age-dependent pathologies.

### Sphingolipids and Insulin Resistance

According to several studies, skeletal muscle accounts for 30–70% of insulin-stimulated glucose disposal in the postprandial state (75, 76). Thus, most of the studies on the mechanisms of insulin resistance induced by lipotoxicity have been carried out mainly in muscle tissue.

In 1963, Randle and colleagues hypothesized that competition between glucose and FA for their oxidation and absorption was responsible for the development of insulin resistance in muscle and adipose tissue (77). *In-vivo* studies in rodents and humans have confirmed a relationship between lipids and insulin resistance, but they also showed that, unlike Randle's hypothesis, lipid-induced insulin resistance was not secondary to inhibition of glycolysis (78). They demonstrated that lipids act directly on insulin signaling, blocking the translocation of the insulin-sensitive glucose transporter GLUT4 to the plasma membrane in response to the hormone, and thus a decrease in glucose uptake in cells (78). In humans, data clearly showed a strong correlation between intramuscular lipid content and insulin cell resistance (79).

Thus, strong evidence exists between muscle lipid accumulation and insulin resistance. However, in some cases, like in the “athlete's paradox,” no correlation between ectopic lipid accumulation and peripheral insulin resistance was found. Athletes display high insulin sensitivity but also present increased levels of intramuscular lipids (80), suggesting that it is not only an increased muscle lipid content that affects insulin sensitivity level, but rather the types of lipids stored in muscle. Indeed, FAs contribute to insulin resistance because they lead to the synthesis of many lipid derivative intermediates such as SL. Ceramide must have been the most studied SL for its critical role in the onset of muscle insulin resistance. However, other SL derivatives synthesized from ceramide (S1P, C1P, SM, and glucosylceramide) were also shown to modulate insulin signaling in muscle cells.

#### Ceramide and Insulin Resistance

One of the first studies to analyse intracellular ceramide concentrations in obese insulin-resistant Zucker fa/fa rats was that of Turinsky et al. (81, 82). They found that these rats had increased muscle ceramide concentrations (81, 82). Increased levels of ceramide have also been detected in skeletal muscle of several other insulin resistance models [insulin resistant rodents, such as ob/ob mice, high fat diet (HFD)-fed mice, and rats infused with intra-lipids] [reviewed in (83)]. Overall, these studies illustrate an inverse relationship between muscle ceramide concentrations and insulin sensitivity. Consistent with data obtained in rodents, human studies also confirm the inverse relationship between an accumulation of skeletal muscle ceramide and insulin sensitivity [reviewed in (84)] (85–87). In addition, the Goodpaster group showed that physical exercise reduced muscle ceramide concentrations in obese and insulin-resistant subjects, all correlated with improved insulin sensitivity (88).

Several studies highlighted the crucial role of muscle ceramide in the development of insulin resistance and type 2 diabetes (T2D) (89). This relationship has also been confirmed *in vitro* in mouse C2C12, rat L6, and human myotubes (90–92). Indeed, the exposure of cells to a saturated fatty acid (SFA) (palmitate) or directly to ceramide inhibited the activation by insulin of glycogen synthesis and glucose transport (89). Overall, these studies demonstrated a strong association between insulin resistance and an increase in ceramide concentrations in skeletal muscles.

*In vivo*, a direct implication of ceramide accumulation in the onset of insulin resistance and diabetes has been established in several studies. Mice fed a HFD and treated with myriocin (inhibitor of the SPT) displayed an inhibition of ceramide synthesis, improvement in muscle Akt phosphorylation, and a better glucose tolerance and insulin sensitivity (93). These data were confirmed in DES1 KO mice. A decrease in ceramide concentrations in insulin-sensitive tissues was observed in dexamethasone-induced diabetes treated mice compared to ceramide concentrations observed in control mice (94). Increased insulin sensitivity was highlighted in the DES1 KO animals compared to their wild-type littermates (94). Finally, treatment with myriocin of HFD-fed rats induced a drop in soleus muscle ceramide concentrations, as well as a normalization of glucose tolerance and insulin sensitivity (95).

However, and despite the abundant literature linking ceramide and muscle insulin resistance, it is important to note that there are nevertheless several studies that do not fully report this causal link. Indeed, some studies did not show any modification of muscle ceramide concentrations in response to lipid overload in rats (96), healthy subjects (97), obese, and diabetic human subjects (98–100). Another study did not show any change in overall muscle ceramide content in T2D patients after endurance training despite a clear improvement in muscle insulin sensitivity (101). In addition, one study showed no difference in muscle ceramide content between T2D patients and healthy individuals (100). Finally, a study showed that a single prior bout of exercise could protect isolated muscle rat against palmitate-induced insulin resistance through a ceramide-independent mechanism (102).

Overall, while numerous studies showed that ceramide was an important player in the onset of muscle insulin resistance, the picture is not that clear-cut, and it cannot be ruled out that other contributors such as diacyl-glycerols [reviewed in (78)] must also play a significant contribution in the appearance of a defect in the action of insulin in muscle.

#### Importance of Ceramide Species in the Development of Muscle Insulin Resistance

Muscle plays a notable role in glucose homeostasis regulation. During obesity, when the buffering action of adipose tissue is saturated and cannot act as a protective metabolic sink, muscle faces an excess of circulating free FA, resulting in an ectopic storage of different lipid such as ceramide in muscle, which are known to induce insulin resistance. Because it remains partially unknown whether specific ceramide species from muscle contribute to insulin resistance and obesity, only recently, generation of different CerS knock-out (KO) mice allowed elucidating their specificities in metabolic disorders.

*In-vitro* studies suggested several ceramide species as key players in mediating deleterious effects of SFAs, such as palmitate on insulin signaling in muscle cells (84). Similarly to what was observed in both L6 and C2C12 myotubes, incubation of primary cultures of muscle cells derived from human biopsies with palmitate led to both an increase of endogenous C16- and C18-ceramide and an impaired insulin signaling (92). Interestingly, these observations were confirmed in primary culture of skeletal muscle cells obtained from diabetic patients (92, 103). Nevertheless, the identity of CerS isoforms implicated in ceramide biosynthesis responsible for muscle insulin resistance remains still elusive.

Each CerS displays characteristic substrate specificity and produces ceramides with specific acyl chain distributions, thereby regulating explicit cell functions. Among them, CerS1 total KO mice suffer from severe neurological modifications (104), while CerS2 KO mice develop hepatocarcinoma (105) and CerS3 KO is lethal (106). CerS4 regulates stem cell homeostasis and hair follicle cycling (107), CerS5 regulates lung phosphatidylcholine synthesis (108), and CerS6 plays a critical role in the development of autoimmune encephalomyelitis (109). These various biological functions regulated by all CerS suggest that ceramide exhibit chain length-specific functions.

Positive correlation between muscle C16- and C18-ceramide content and insulin resistance had been highlighted for some time (95, 110–112). However, molecular evidences of an intramuscular synthesis of these ceramide species related to their insulin resistant properties were still lacking. In the last few years, generation of CerS KO mice allowed to go further in the identification of which ceramide species is (are) involved in the development of muscle insulin resistance.

In 2016, Gosejacob et al. demonstrated that CerS5 played a central role to maintain C16-ceramide pool in insulin sensitive tissues (113). They showed that CerS5 expression was essential to maintain cellular C16-ceramide content in lung, spleen, muscle, white adipose tissue (WAT) and liver, independently of feeding conditions. Under HFD conditions, a massive increase in C16-ceramide content was mainly observed in WAT compared to control mice (113). CerS5 deficiency prevented WAT C16-ceramide increase and improved glucose intolerance, insulin resistance, WAT mass, adipocyte size, and triglycerides levels in mice challenged with HFD (113). Altogether, this study highlighted new insight about the implication of CerS5 in maintaining whole-body homeostasis, but independently from its expression in muscle, suggesting that C16-ceramide produced by CerS5 may not play a role in the onset of muscle insulin resistance. In agreement, Turpin-Nolan et al. (114) found that mice with a muscle-specific CerS5 deletion exhibited no alterations in adiposity, systemic insulin sensitivity, and glucose tolerance upon exposure to a HFD.

A study investigated a role for C16-ceramide produced by CerS6 (using CerS6 KO mice) in the progression of muscle insulin resistance. CerS6 is another CerS isoform known to generate ceramide with C16 acyl chain, and HFD-fed CerS6 KO were protected from HFD-induced obesity and glucose intolerance. Furthermore, mice exhibited reduced C16-ceramide content in liver, white and brown adipose tissues but not in muscle (115). Interestingly, mice with a muscle-specific CerS6 did no reverse the effect of HFD on insulin sensitivity (114). Altogether, these studies argue against a role of a direct role of a local production of C16-ceramide either by CerS5 or CerS6 into muscle insulin resistance.

**Figure 3:**
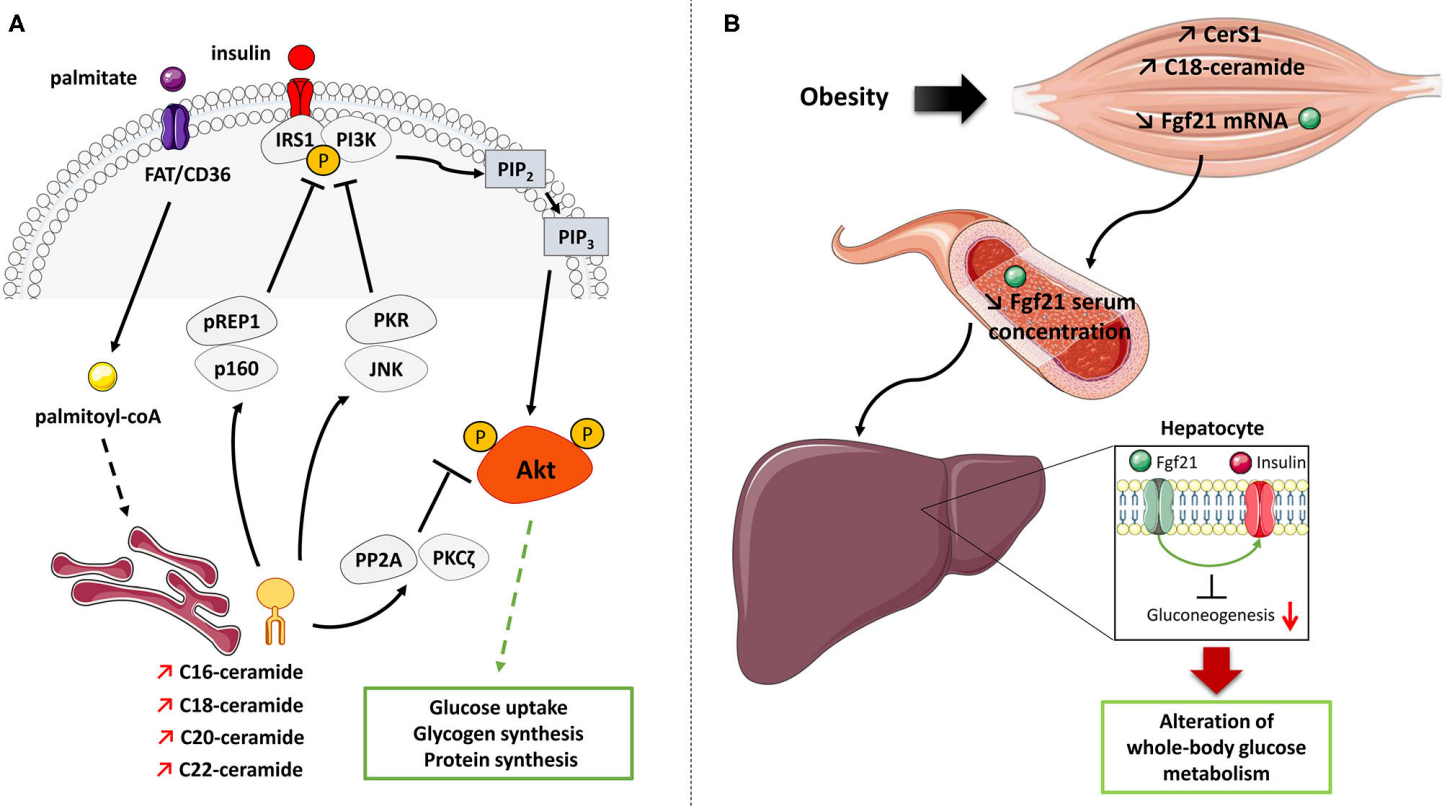
Mechanisms of action of ceramide on insulin signaling and glucose metabolism in muscle cells. **(A)** Palmitate induces the *de-novo* production of several ceramide species in muscle cells. Ceramide inhibits the muscle insulin signaling pathway and muscle metabolism by targeting two important actors, Akt and IRS1. Ceramide rapidly activates either PP2A or PKCζ to inactivate Akt. Ceramides also induce PKR/JNK and/or Prep1/p160 axes to target IRS1. IRS1, Insulin receptor substrate 1; JNK, c-Jun NH2-terminal kinase; PI3K, Phosphoinositide-3-kinase; PKCζ, Protein kinase C ζ; PKR, Double stranded ARN-activated protein kinase; PP2A, Protein phosphatase 2A; Prep1, Pbx regulating protein 1; P, phosphorylation. **(B)** Proposed model of muscle ceramide action on regulating whole-body glucose metabolism. Obesity-induced lipid overflow in muscle induces an increase in CerS1 expression and C18-ceramide content which decrease in Fgf21 production and secretion from muscle cells. This lack of Fgf21 secretion could affect the ability of insulin to suppress hepatic glucose production and will induce hyperglycemia.

Given the lack of molecular evidence for a role of intramuscular C16-ceramide production by these two CerS in muscle insulin resistance, other studies focused on C18-ceramide as another potential candidate. Gosejacob et al. (113) already showed that CerS5 KO mice challenged with a HFD were protected against C18-ceramide accumulation in muscle. These data, in conjunction with other studies showing that C18-ceramide content in muscle was increased with lipotoxicity (92, 103, 114), suggest that accumulation of this ceramide species may play a role in muscle insulin resistance appearance (Figure 3A). Additional studies performed in rats (95, 116) and humans (117) also highlighted a direct role of C18-ceramide in the onset of muscle insulin resistance.

Synthetized by CerS1, C18-ceramide is one of the major ceramide species upregulated in skeletal muscle of mice fed with HFD (113, 114). Turpin-Nolan et al. (114) generated a global CerS1 KO mice that, under a HFD, displayed reduced C18-ceramide production in skeletal muscle, accompanied by an increase in C16-, C22:0, C22:1, C24:0, and C24:1 ceramide species. Particularly, CerS1 deficiency did not affect the SL content in heart, liver, and white adipose tissue (114). These changes in ceramide contents were accompanied with decreased body weight and adiposity and higher energy expenditure and a better glucose tolerance (114). In addition, CerS1 deficient mice improved their glucose tolerance and insulin sensitivity under HFD (114). Mice lacking CerS1 were also generated specifically in skeletal muscle (114). These mice under a HFD displayed a strong reduction in C18-ceramide in this tissue, but, and in opposite to global CerS1 KO mice, no difference in weight gain, adiposity, and food intake was observed (114). However, glucose metabolism was still improved in muscle CerS1 deficient mice under HFD in response due to an increase of Fibroblast Growth Factor 21 (Fgf21) release from skeletal muscle into the circulation (114). Fgf21 is a growth factor mainly synthetized by the liver and other organs, and it can be secreted as a compensatory mechanism to fight against metabolic disorders associated with obesogenic conditions, and is known to exert anti-inflammatory and insulin-sensitizing actions (118). In absence of CerS1 in muscle, muscle-derived Fgf21 seemed to improve the ability of insulin to suppress hepatic glucose production (114) (Figure 3B). Taken together, these studies highlighted CerS1 and its consequent reduction of C18-ceramide in muscle as key components in ameliorating whole-organism glucose metabolism. However, the latter study also reveals that intramuscular C18-ceramide produced by CerS1 may not play a direct role in muscle insulin resistance (114).

Beyond genetic models, a specific CerS1 pharmacological inhibitor, P053, has been generated (119). Its daily oral administration in mice fed an HFD decreased by 50% C18-ceramide content in skeletal muscle. This decrease was associated with reduced whole-body adiposity due to enhanced FA oxidation in this tissue. However, and unlike the CerS1 muscle deficient mice, this inhibitor did not improved glucose tolerance and insulin sensitivity in HFD-fed mice (119). This suggests that C18-ceramide may not be the main SL lipid actor for the development of insulin resistance, or possibly that the residual muscle C18-ceramide content (50%) was sufficient to inhibit insulin sensitivity (in opposite to 90% ablation observed in Turpin-Nolan's study). Interestingly, another study tended to suggest that other ceramide species could also be involved in muscle insulin resistance. Bandet et al. (120) showed that overexpression of CERT transporter in in tibialis anterior muscle from mice fed a HFD induced a decrease in concentrations of C16-, C22-, C24:1-, and C24-ceramide species associated with an improved insulin response. No change in both C18- and C20-ceramide species was observed (120). Thus, further studies will be necessary to exactly define whether other ceramide species than C18-ceramide produced by others CerS (i.e., CerS3 and 4) could also play a significant and deleterious role in the modulation of muscle insulin sensitivity.

#### Subcellular Localization and Mechanism of Action of Ceramide in Muscle Cells

Subcellular localization of SL has emerged to be crucial in relation to insulin resistance in skeletal muscle. Ceramide is generated in the ER through *de-novo* synthesis and is transported to the Golgi apparatus to be metabolized into other SL (Figure 1). This transport involves an ATP-dependent carrier called CERT (121). Ceramide can also be produced in the plasma membrane through conversion of SM into ceramide by SMase (Figure 1) (4). Interestingly, CerS isoforms were found in various intracellular organelles, such as the ER (122), the mitochondria (123), the nucleus (124), and the Golgi apparatus (123), suggesting a possible local ceramide production in all these compartments. Thus, contribution of ceramide to cellular dysfunctions could be related to these subcellular compartments.

Perreault et al. (125) found an accumulation of ceramide, sphingomyelin, and lactosylceramide in sarcolemma of insulin-resistant individuals. Similar data were observed by Chung et al. (112). Interestingly, association between raise in intramyocellular ceramide concentrations and hyperinsulinemia/hypertriglyceridemia was only limited to the sarcolemmal fraction (112), and only C16- and C18-ceramides were found to be positively correlated with insulin resistance markers (112). These data should be closely compared to the absence of role of CerS1, 5, and 6 isoforms in muscle insulin resistance found in some studies (113, 114, 119). In these latter studies, both C16 and C18-ceramides were quantified in whole muscle. Therefore, it would be important to determine whether these CerS could affect specifically subcellular localization of C16 and C18-ceramides.

In sarcolemma compartment, ceramides have been shown to inhibit insulin signaling through different mechanisms in muscle cells. The first step in activating the insulin signaling pathway is the hormone binding to its membrane receptor, leading to the autophosphorylation of its β subunits (126). Following this autophosphorylation, the insulin receptor catalyzes the activation of insulin receptor substrates (IRS). Six different isoforms of IRS are expressed, but only IRS1 and 2 control insulin signal transduction. IRS1 is predominantly expressed in skeletal muscle where it plays a crucial role in the transmission of the insulin signal (127). Activated IRS1 activates a protein called PI3K that generates specific membrane phospholipids essential for the activation of a protein kinase called Akt (also called Protein Kinase B) (126, 128). In response to insulin, Akt is recruited to the plasma membrane via phospholipids generated by PI3K where it is activated by phosphorylation at two critical sites: threonine 308 (T308) in the activation loop and serine 473 (S473) in the hydrophobic motif of the kinase (126). The phosphoinositide-dependent kinase 1 (PDK1) phosphorylates Akt at its T308 site and the S473 site is phosphorylated by the mTORC2 (mammalian target of rapamycin-2) complex (126). Akt relays the many metabolic actions of insulin in its different target tissues (128, 129). Insulin stimulates the uptake of glucose in skeletal muscles and adipocytes, induces the synthesis of glycogen and its storage in muscle and in the liver, induces protein synthesis in muscle, and inhibits adipocyte lipolysis and production hepatic glucose (89, 130).

The mechanisms by which ceramide acts on the insulin signaling pathway in insulin-sensitive tissues are now clear, at least in both rodent and human muscle cells. In these cells, ceramide targets the insulin signaling by specifically inhibiting in a time-dependent manner two specific actors in this pathway: Akt and IRS1. Regarding Akt, ceramide rapidly (within 2 h) activates the atypical protein kinase C ζ (PKCζ), which interacts and phosphorylates the pleckstrin homology (PH) domain of Akt on a Thr34/Ser34 residue, thus inducing its sequestration in specialized areas of the plasma membrane called Caveolin Enriched Microdomains (CEM) (131–136). Targeting Akt in CEM prevents the kinase from being recruited to the plasma membrane where it is normally activated in response to insulin (136, 137). In this time frame, the authors showed that the inhibition of the insulin signaling pathway was not mediated at the level of the insulin receptor, IRS1, PI3K, or PDK1, but was only due to a targeted action of ceramide on Akt (91, 138, 139). In CEM devoid cells, ceramide inhibits Akt by a mechanism involving the protein phosphatase 2A (PP2A) (90, 137). In the longer term, ceramide also target and inhibit IRS1 by mechanisms involving PKR/JNK (double-stranded RNA-dependent protein kinase/c-Jun kinase) and/or Prep1/p160 (Figure 3A) (140, 141).

*De-novo* synthetized ceramides are transported from the ER to the Golgi apparatus through a transporter called CERT, where they are metabolized into SM (4). Disrupted CERT-mediated transport of ceramide from the ER to the Golgi was observed in both mouse and human muscle cells under lipotoxic conditions, leading to a decrease in Golgi-based SM synthesis (120). Interestingly, in T2D patients, CERT expression was found to be downregulated in muscle (142), suggesting that, in addition to the increased *de-novo* ceramide biosynthesis observed in the ER, a reduced transport of ceramide due to decreased levels of CERT could play a role in muscle insulin resistance. However, how accumulation of ceramide in the ER through CERT inhibition plays a role in insulin resistance remains elusive.

The ER has been shown to be the major *de-novo* production site of ceramide during lipotoxicity, and ceramide accumulation in this compartment can induce changes in the ER homeostasis, and a durable unfolded protein response called ER stress (143). Long-term ER stress has been observed in obesity and insulin resistance and is associated with cellular dysfunction in liver and adipose tissue (144, 145). In muscle cells, however, activation of ER stress in response to ceramide accumulation remains mild (146), and does not play a major role in the onset of insulin resistance (146, 147). These data could suggest that ER-produced ceramide must somehow transfer to the plasma membrane, maybe through specialized membrane contact sites between these two compartments, to exert their negative effects on insulin signaling.

Another possibility could be related to the accumulation of ceramide in the mitochondria. Indeed, mitochondria-based ceramide has also been observed (148) where they have been shown to inhibit mitochondrial fatty acid oxidation in liver and brain (115, 125, 149). Similar observations have been made in muscle mitochondria where addition of C2-ceramide to isolated mitochondria inhibited mitochondrial respiratory chain function (150). More recently, Perreault et al. showed that ceramide or dihydroceramide inhibited complex 1 and 3 of the electron transport chain (125), demonstrating a possible and important role for these lipids in the appearance of an oxidative stress, systemic inflammation and insulin resistance. Interestingly, disruption of mitochondria-associated ER membrane (MAM) integrity resulted in muscle insulin resistance in both mice and humans (151). Ceramide metabolism and especially CerS were located in MAM from liver (152), supporting the hypothesis that obesity could increase ceramide levels in MAM and could contribute to muscle insulin resistance.

Overall, these observations suggest that mechanisms directing the negative action of ceramide on muscle insulin sensitivity are rather complex, and may imply to consider SL subcellular localization in order to fight insulin resistance.

#### Ceramide Lipid Derivatives and Muscle Insulin Resistance

**Figure 4:**
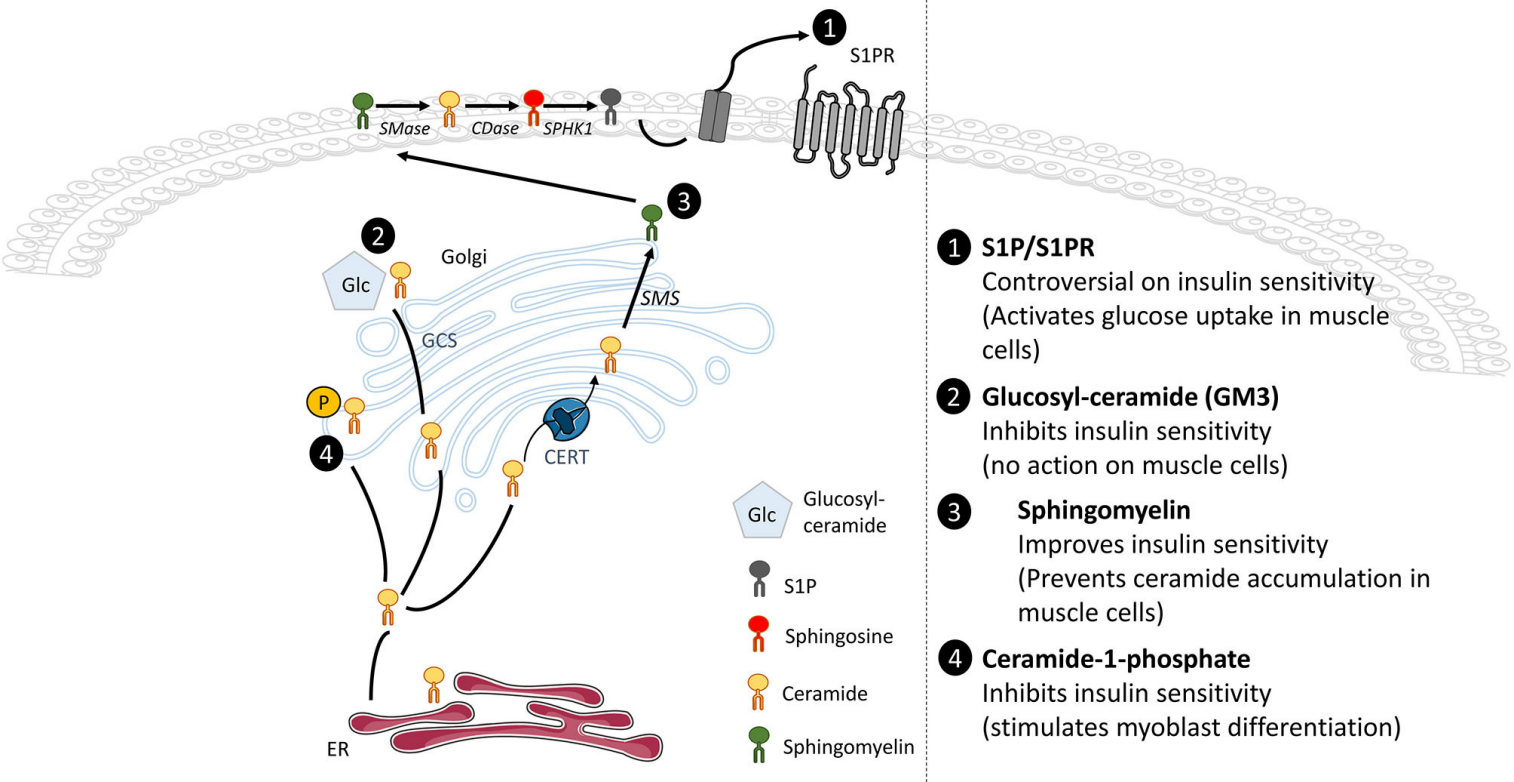
Effects of ceramide metabolite accumulation on insulin signaling. Whole-tissue accumulation of ceramide-1-phosphate and glucosylceramide damages insulin sensitivity without significant action in muscle glucose metabolism. In opposite, accumulation of SM prevents ceramide buildup and improves insulin sensitivity in muscle cells. The SphK1/S1P/S1PR axis displays controversial actions on insulin sensitivity but activates glucose uptake in muscle cells.

Several other SL derivatives synthesized from ceramide have also been shown to regulate insulin signaling in muscle cells (Figure 4).

##### Sphingosine-1-phosphate

Recent evidence suggests that obesity also increased other SL content such as S1P in muscle. Hu et al. determined that lipotoxicity positively increased S1P levels through upregulation of SphK1 activity in C2C12 myotubes, but also in isolated skeletal muscle from HFD-fed mice (153). These data suggested for the first time that both S1P and SphK1 could play a role in metabolic diseases such as obesity or T2D (153). Interestingly, Rapizzi et al. reported a crosstalk between S1P and insulin signaling in C2C12 myoblasts as they showed that S1P, through the transactivation of the insulin receptor, increased glucose uptake in muscle cells (154).

These data considered, successive studies wondered whether the muscle SphK/S1P axis could be implicated in modulating insulin signaling in a lipotoxicity context observed in obesity and T2D. Several studies highlighted a critical role of SphK1 in the regulation of the whole body glucose homeostasis (155, 156). HFD-fed SphK1 KO mice exhibited high fasting glucose levels and severe glucose intolerance compared to their WT HFD-fed littermates. However, this was not associated with an increase of the global insulin resistant state induced by obesity, but rather with a failure of compensatory insulin production by pancreatic β-cells in HFD-fed SphK1 KO mice (156). In fact, HFD-fed SphK1 KO mice displayed a significant reduction of β-cells mass, consequence of an increased β-cells apoptosis in comparison with HFD-fed WT mice. These data highlighted that SphK1 played a key role in regulation whole-body glucose homeostasis under lipotoxic conditions (156). However, whether muscle SphK1 expressed exerted a role in enhancing whole-body glucose homeostasis remained unclear. Bruce et al. (155) were pioneers to identify a role of muscle SphK1 in the etiology of obesity and insulin resistance. HFD-fed mice overexpressing SphK1 displayed increased SphK1 activity in skeletal muscle, were protected against ceramide accumulation and displayed an improved insulin-stimulated glucose uptake (155). In opposite, inhibition of SphK1 activity strengthened the deleterious effect of palmitate on insulin-stimulated glucose uptake and further reduced the activity of insulin signaling proteins in L6 myotubes (157). Furthermore, treatment of HFD-fed mice with FTY20, a S1P analog, improved whole-body glucose tolerance, reduced muscle ceramide content and increased muscle insulin-stimulated glucose uptake and Akt phosphorylation (158). Taken together, these results suggest that, under lipotoxic conditions, muscle SphK1 could play a protective role against the onset of insulin resistance.

However, genetic (SphK1 KO) or pharmacological (SphK1 inhibitor) approaches displayed improved glucose tolerance and insulin sensitivity, and lowered fasting glucose levels in obese mice (159). In addition, improved insulin-dependent phosphorylation of Akt was observed in adipose tissue, liver, and muscle of HFD-fed SphK1 KO mice. The discrepancy between these data and Bruce's could come from the diet they used throughout the studies (159, 160). The first study used a fructose-associated HFD to challenge pancreatic β-cell function in an obesity context, whereas the second study used a simple HFD to induce obesity. Altogether, these studies highlight a possible divergent role of the muscle SphK1/S1P axis on regulating whole-body glucose homeostasis in mice. Both studies used a global SphK1 KO approach, and further analyses of a muscle-targeted deletion of SphK1 would be more conclusive.

In opposite to SphK1, muscle SphK2 overexpression had no effect on muscle lipid content, suggesting that SphK2 did no regulate muscle ceramide levels (155). More recently, Ravichandran et al. (161) aimed to better understand the role of SphK2 in glucose and lipid metabolism. They characterized metabolic parameters of SphK2 KO old mice compared to control old mice, as aging is associated with weight gain and obesity (161). SphK2 KO mice seemed to be protected against age-related changes of adipose tissue as they displayed decreased weight and fat mass and increased lean mass (161). Moreover, old SphK2 KO mice saw their glucose tolerance and insulin sensitivity improved with aging compared with WT mice (161). These enhanced metabolic e?ects could be the result of increased adiponectin plasma levels and enhanced lipolysis (161). It still remains to know whether muscle insulin sensitivity was improved these conditions. Nevertheless, this study indicates that SphK2 may be a negative regulator of glucose homeostasis in an obesity context. Interestingly, a role of SphK2 in promoting β-cell lipotoxicity has also been demonstrated as SphK2 deficiency prevented β-cell mass loss in lipotoxic conditions (162). Overall, these data indicated that inhibition of SphK2 could be an attractive target to develop treatments to ameliorate insulin resistance but also insulin secretion associated with obesity and T2D.

A small number of studies have also suggested the involvement of additional SL derivatives as being able to modulate glucose homeostasis. However, their direct action in muscle cells is not always well defined and clear (Figure 4).

##### Ceramide-1-phosphate

A study has shown that the total silencing of CerK, ceramide kinase responsible for the formation of C1P from ceramide, protected animals from obesity and glucose intolerance induced by a HFD. In addition, CerK silencing also protected mouse adipose tissue from macrophage infiltration, thereby preventing adipose tissue inflammation of (163). At the muscle level, C1P stimulated myoblast proliferation via a lysophosphatidic acid signaling axis (164), in a PI3K, Akt, ERK 1/2, and mTOR dependent manner (165). This suggests that C1P could be involved in the regulation of muscle regeneration and repair.

##### Sphingomyelin

A metabolomic study demonstrated that reduced levels of plasma C16:1-SM species were predictive of T2D (166). In addition, inhibition of SMase in muscle cells induced an increase in ceramide content and impairment of insulin signaling (167). Obese individuals with glucose intolerance showed increased muscle ceramide content and lower muscle SM compared with obese individuals with normal glucose tolerance (168). Overall, these data suggest that SM biosynthesis from ceramide may be protective for maintaining insulin sensitivity, and that inhibition of SM synthesis could have serious consequences on cell insulin sensitivity. A study reinforced this hypothesis, as a pharmacological inhibition of SMS2 in C2C12 myotubes induced ceramide accumulation in cells and an alteration of insulin signaling (167). In addition, Bandet et al. showed in muscle cells that increased concentrations of ceramide in response to palmitate were associated with a malfunctioning transport of the SL from the ER to the Golgi apparatus, consequence of a defected expression of CERT, and a concomitant reduced SM biosynthesis (120). *In-vitro* overexpression of CERT in muscle cells under lipotoxic conditions, or *in vivo* in the anterior tibialis muscle of HFD-fed mice improved insulin sensitivity (120), demonstrating that transformation of ceramide into SM improved muscle insulin sensitivity.

##### Gangliosides

Inhibition of glycosphingolipid synthesis significantly improved insulin sensitivity and glucose homeostasis in Zucker diabetic fatty rat, *ob/ob* mice, and HFD-fed mice (169, 170). The involvement of a particular ganglioside, GM3 (ganglioside monosialo 3) was particularly highlighted in this process. TNFα-induced insulin resistance was shown to be associated with high concentrations of GM3 in adipocytes (171), and GM3 synthase deficient mice fed a HFD displayed a better glucose tolerance than HFD-fed control mice (172). In humans, serum GM3 concentrations were increased in T2D patients (173), and they are associated with risk factors of metabolic syndrome (174). In skeletal muscle, GM3 content was significantly higher in T2D rats compared with control rats, but significantly lower in T1D rats compared with their controls (175). However, involvement of GM3 in inhibiting insulin action in muscle cells remains elusive since a direct addition of GM3 or GCS overexpression failed to inhibit insulin signaling in C2C12 myotubes (176). In addition, glucosylceramide concentration went down in muscle of mice fed a HFD, and GSL accumulated in adipocytes, where it exerted negative effects on insulin signaling (176).

Overall, these ceramide derivatives could therefore play an important role in modulating muscle insulin sensitivity, but crucial lack of human data on these SL as regulators of muscle metabolism prevents definitive conclusions.

## Sphingolipids and Physical Activity

Several studies attempted to investigate whether a correlation existed between physical exercise, known to improve cell insulin sensitivity in diabetic patients (177), and intramyocyte levels of ceramide, acknowledged to be harmful to insulin sensitivity (see previous chapter). The rationale for this question was whether physical exercise, in addition to improving muscle uptake of glucose by an insulin-independent pathway (178), could prevent ceramide from accumulating in muscle cells, and thus resensitize them to the action of insulin.

A pioneering study carried out in 2004 in humans showed that, in opposite to what was expected, acute physical exercise (3 h bicycle exercise) induced an increase in muscle concentrations of ceramide (+ 39%) (179). Another study performed by the same group went more in detail and assessed SL content in rats exercised for 30 min, 90 min, and until exhaustion (180). The authors showed that exercise gradually increased muscle ceramide levels (+27%) in two different muscles types (soleus and red section of gastrocnemius). Surprisingly, in white section of gastrocnemius, ceramide content decreased progressively until the 90th minute of exercise and returned to control value when mice were exhausted (180). Dihydrosphingosine content was also assessed. It increased gradually over time in the three muscle types to reach maximum level (80 to 100%) at the point of exhaustion (180). Levels of sphingosine rapidly increased by 20–30% after 30 min of running and stayed at that level until exhaustion (180). S1P levels stayed stable until 90th minute of running and reached a twofold increase at the point of exhaustion in soleus. In red and white sections of the gastrocnemius, S1P levels went up and down throughout physical exercise (180). Circulating S1P levels were also increased in response to acute (60 min) physical exercise (181). Activities of key enzymes involved in ceramide metabolism (SPT, nSMase, aSMase, CDase) were also affected by exercise. Thus, SPT activity went up gradually with the duration of the exercise, while nSMase activity did not move, and both aSMase and CDase activities went down (180). These data indicate that the exercise strongly affected the activity of the key enzymes involved in ceramide metabolism and increased ceramide *de-novo* synthesis.

**Figure 5:**
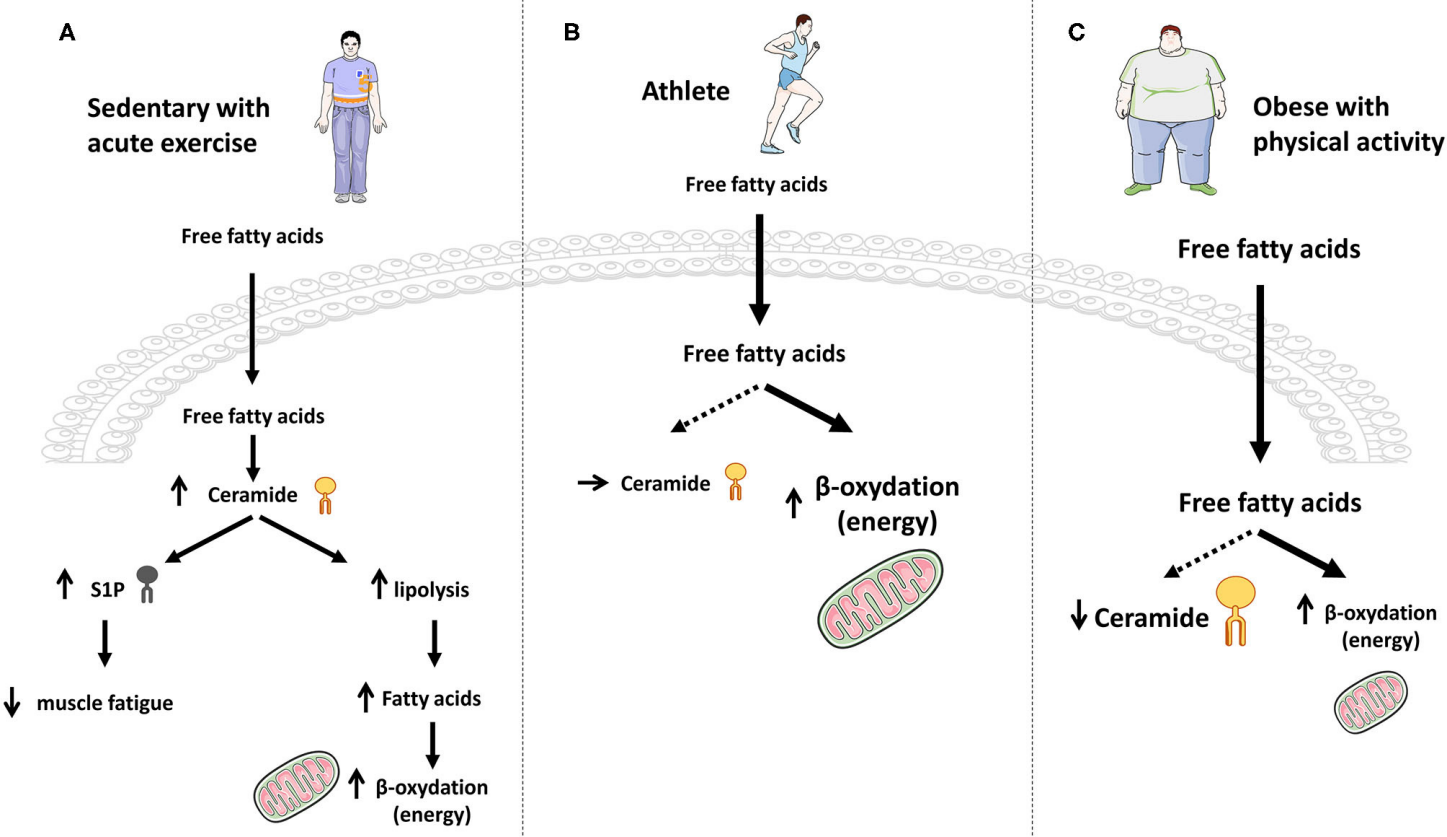
Effect of exercise on ceramide accumulation and muscle energy requirements. **(A)** In sedentary lean people, acute exercise induces a transient increase in ceramide synthesis that momentarily blocks the anti-lipolytic action of insulin and therefore favors FA production and energy needs through β-oxidation. **(B)** Chronic exercise promotes increased entry of FA into muscle cells and their oxidation to fulfill the energy need, without any ceramide production. **(C)** In obese patients practicing physical activity, large intracellular FA concentrations are deflected toward β-oxidation for energy purposes, thereby reducing muscle ceramide synthesis flux and concentrations.

In an attempt to explain these results, the authors advanced the hypothesis that the increase in plasma concentrations of free FA (lipolysis), secondary to physical exercise (182), could be favorable to the increase of the *de-novo* synthesis of ceramide. It is also possible that acute exercise-induced ceramide accumulation could be the consequence of an increased inflammatory response to exercise. Furthermore, S1P accumulation could prolong exercise capacity and delay muscle fatigue (Figure 5A) (180). Indeed, studies also showed an increase in muscle S1P content with exercise (181), and that S1P improved muscle strength, mass, and metabolism (59, 183–185). These data were confirmed in a recent human study where they found that total muscle ceramide and other SL species concentrations were significantly increased by acute exercise (2 h), and then rapidly returned to basal after 2 h resting (117).

The role of this increase in muscle ceramide content during acute physical exertion remains currently unknown, but we can hypothesize that it would block insulin action and boost the use of lipids as an energy source for physical exercise. In opposite, increased S1P could exert beneficial effects on the promotion of fatigue resistance.

Endurance training studies gave different results. No modification or even a decrease in the concentrations of intramuscular ceramide was observed in rats trained for 6–8 weeks (wheel exercised) compared to sedentary rats (186, 187). Results of these studies were confirmed in humans where ceramide concentrations in skeletal muscles of non-obese sedentary volunteers and non-obese endurance-trained male athletes (aerobic exercise sessions) were measured, and no difference was observed (188, 189). Similar observations were obtained in lean women (Figure 5B) (190).

In contrast, ceramide concentrations measured in obese and T2D patients who were sedentary or had practiced moderate but prolonged physical activity were found to be decreased in response to exercise training (190–195). Interestingly, Bruce et al. showed that C16-, C16:1-, C18-, C18:1-, C18:2-, and C20-ceramide concentrations were decreased (191), and this study also showed that physical exercise increased the activity of CPT1, thus pushing FAs toward mitochondria oxidation and diverting them from ceramide synthesis (191). Even if this latter study suggests that physical exercise could inhibit ceramide to be synthetized *de novo* in obese and T2D patients, more work will be necessary to figure out whether CerS expression was modified by exercise, and to exclude an exercise-induced inhibition of either the SMase or the salvage/recycling pathway in this process (Figure 5C).

AMPK could play an important role in the inhibition of ceramide synthesis observed in skeletal muscle in response to exercise in T2D patients. Indeed, utilization of metformin, a ubiquitously prescribed anti-diabetic drug that was described to act through AMPK activation (196), improved insulin sensitivity at both systemic and muscle levels by augmenting Akt phosphorylation and decreasing ceramide content (116). Direct involvement of AMPK in this process was further characterized after treatment of C2C12 muscle cells with 5-amino-1-β-D-ribofuranosyl-imidazole-4-carboxamide (AICAR), a well-established AMPK activator. AICAR treatment prevented completely palmitate-induced ceramide content in cells (197), suggesting that AMPK activation could be an important exercise-associated mechanism to decrease muscle ceramide content and to improve metabolic functions in T2D patients.

In summary, physical exercise seems to exert opposite actions on ceramide metabolism, depending on the chronicity of the effort (Figure 5):

- an acute action inducing a transient increase of the *de-novo* ceramide synthesis, thus promoting muscle FA β-oxidation by temporarily blocking the anti-lipolytic action of insulin.
- a more long-term action in lean, obese and T2D individuals where lipids are deflected toward an increased β-oxidation for energy purposes, thereby reducing muscle ceramide biosynthesis flux (no need to block insulin action). Interestingly, chronic exercise-induced FA β-oxidation decreases FA conversion to ceramide, hence improving muscle sensitivity to insulin in obese/T2D patients.

## Circulating Sphingolipids

Negative consequences of tissue-based ceramide (and some ceramide metabolites) accumulation have now been extensively highlighted over the years, but it looks now apparent that circulating SL also play a distant and additional role on insulin-sensitive organs such as skeletal muscle. Indeed, several studies have reported an increase in circulating ceramide content in the plasma of obese and T2D mice (94, 198, 199), as well as with insulin resistance in obese people with T2D (200, 201). Interestingly, weight loss induced by gastric bypass surgery or changes in lifestyle produced a decrease in plasma ceramide content in patients (199, 202–206). Importantly, strong correlations have been established between plasma ceramide concentrations and insulin resistance and inflammation (200, 201, 207, 208). It is interesting to note that one study has also shown that serum levels of the ceramide-derived ganglioside GM3 were also increased in T2D patients (173).

A possible and direct role of circulating ceramide in the development of skeletal muscle insulin resistance has been proposed by Boon et al. (199) a few years ago. They showed that ceramide concentrations were elevated specifically in low-density lipoproteins **(**LDL) of T2D patients compared with insulin-sensitive individuals, independently of obesity (199). Interestingly, they demonstrated that LDL-ceramide caused whole-body insulin resistance in lean mice, an effect mediated by a decrease of insulin signal in skeletal muscle. *In vitro* incubation of L6 muscle cells with C24:0-ceramide-enriched LDL induced a loss in insulin response, demonstrating that LDL-ceramide were taken up by muscle cells and were directly affecting muscle insulin signaling (199). Secretion of ceramide into LDL lipoproteins by the liver into the circulation had already been shown long ago (209, 210), suggesting that, in addition to muscle-based *de-novo* synthetized ceramide, hepatocyte-produced ceramide can also affect remotely muscle insulin sensitivity, further complicating possible strategies envisaged to combat the deleterious peripheral action of ceramide on muscle insulin sensitivity.

Like ceramide, S1P can also be secreted into the bloodstream associated to high-density lipoproteins (HDL). Circulating S1P is mainly derived from erythrocytes and vascular endothelial cells in healthy subjects (211). However, plasma levels of S1P were found to be higher in different rodent models of insulin resistance (212), in obese patients compared to non-obese individuals (213), and correlated with HOMA-IR, HbA1c, and body mass index (212). In the diabetic state, S1P binds physically to apolipoprotein M (ApoM) and is excreted by the liver inside ApoM-containing HDL vesicles into the plasma (214–216). However, S1P is a key component in the antiatherogenic properties of HDL (217, 218), and two very recent studies just showed that apoM/S1P exerted a protective role against the development of insulin resistance. Indeed, Kurano et al. showed that deletion of apoM in mice was associated with a degradation of insulin sensitivity associated with a decreased Akt phosphorylation in skeletal muscle (219). In opposite, overexpression of apoM induced an improvement of insulin sensitivity in mice and enhanced muscle Akt phosphorylation (219). Identical results were observed in Goto-Kakizaki Rats (220). Altogether, these studies demonstrate that circulating S1P can exert protecting effects against the development of tissue insulin resistance.

Quantification of circulating SL could also be important in the context of monitoring the development of diabetes since it has recently been shown that certain SL could be used as biomarkers to identify individuals at risk of developing T2D. Indeed, it has been demonstrated that circulating concentrations of Dcer were significantly elevated in insulin-resistant individuals with T2D (221), and more importantly, in pre-diabetic individuals up to 9 years before the onset of the disease (222). Interestingly, circulating Dcer were better biomarkers of TD2 than circulating ceramides (223). Few data, at the cellular level or in animals, exist on the mechanistic role of Dcer on the pathophysiology of T2D. In addition, no data are available about the origin of these circulating Dcer, and about the carrier (lipoproteins, exosomes…) they are using in blood circulation.

Interestingly, 1-deoxysphinganine plasma levels were also found to be increased in non-human primates maintained on a HFD (224). 1-deoxysphinganine is produced through the condensation of alanine rather than serine with palmitoyl-CoA by SPT (7), suggesting that HFD feeding could increase the utilization frequency of alanine by SPT. Levels of 1-deoxysphinganine correlated with HOMA-IR, indicating that elevated plasma deoxysphingolipids could also be biomarkers of insulin resistance (224).

Thus, additional studies need to be carried out to clarify the interest of these ceramide-derived SL species as biomarkers or as therapeutic targets in T2D.

## Conclusion

SL influence several important processes in skeletal muscle, from differentiation, regeneration, and fatigue to contraction and insulin response. SL metabolism is quite complex and depends on numerous enzymes and signaling pathways, and important roles of some SL metabolites, especially those of ceramide and S1P, in the regulation of physiological or pathophysiological skeletal muscle phenomena begin to be really highlighted. The discovery of the different pathways of ceramide intermediary metabolism has provided the field with a number of exciting targets in order to manipulate SL levels. As such, pharmacological and genetic strategies that could modulate levels of muscle SL should lead to beneficial effects in the progression and development of muscle disorders, thus supporting new approaches for potential treatments of muscle physiopathology.

## Author Contributions

EH: conceived the idea. ST-C, JG, OB, HL, and EH: wrote the manuscript and critically reviewed the manuscript and figures. ST-C: figure preparation. All authors: approved the final manuscript.

## Conflict of Interest

The authors declare that the research was conducted in the absence of any commercial or financial relationships that could be construed as a potential conflict of interest.

## Abbreviations

ACER: Alkaline ceramidases
ApoM: Apolipoprotein M
ASAH1: Acid ceramidase
ASAH2: Neutral ceramidase
aSMase: Acid sphingomyelinase
C1P: Ceramide-1-phosphate
CDase: Ceramidase
CEM: Cavolin Enriched Microdomains
CerK: Ceramide kinase
CerS: Ceramide synthases
CERT: CERamide Transporter
CGT: Galactosyl transferase
CPT1: Carnitine palmitoyltransferase 1
Dcer: Dihydroceramide
DES1: Dcer desaturase-1
DES2: C4-hydroxylase / Δ4-desaturase
ER: Endoplasmic reticulum
ERK1/2: Signal-regulated kinase ½
GCS: Glucosylceramide synthase
GBA: Acid β-glucosidase
GLUT: Glucose transporter
GM3: Ganglioside monosialo 3
FA: Fatty acids
FABPm: Plasma membrane-associated FA-binding protein
FAT/CD36: FA translocase/cluster of differentiation
Fgf21: Fibroblast Growth Factor 21
HFD: High fat diet
IRS: Insulin receptor substrates
JNK: c-Jun kinase
KDSR: 3-keto-dihydrosphingosine
KO: Knock-out
LDL: Low-density lipoproteins
LPP: Phosphohydrolases
mTORC2: Mammalian target of rapamycin-2
nSMase: Neutral sphingomyelinase
PDK1: Phosphoinositide-dependent kinase 1
PI3K: Phosphoinositide-3- kinase
PKCζ: Protein kinase C ζ
PKR: Double-stranded RNA-dependent protein kinase
PP2A: Protein phosphatase 2A
Prep1: Pbx regulating protein 1
S1P: Sphingosine-1-phosphate
S1PR: S1P receptors
SFA: Saturated FA
SMS: SM synthases
SL: Sphingolipids
SM: Sphingomyelin
SMase: Sphingomyelinases
SMS: Sphingomyelin synthase
SphK: Sphingosine kinases
SPL: S1P-lyase
SPT: Serine palmitoyl transferase
T2D: Type 2 diabetes
TAG: Triacylglycerol
TNFα: Tumor necrosis factor α
WAT: White adipose tissue.
